# Mainzelliste SecureEpiLinker (MainSEL): privacy-preserving record linkage using secure multi-party computation

**DOI:** 10.1093/bioinformatics/btaa764

**Published:** 2020-09-01

**Authors:** Sebastian Stammler, Tobias Kussel, Phillipp Schoppmann, Florian Stampe, Galina Tremper, Stefan Katzenbeisser, Kay Hamacher, Martin Lablans

**Affiliations:** Department of Computer Science, Technische Universität Darmstadt, 64289 Darmstadt, Germany; Department of Computer Science, Technische Universität Darmstadt, 64289 Darmstadt, Germany; Department of Computer Science, Humboldt-Universität zu Berlin, 10099 Berlin, Germany; Federated Information Systems, German Cancer Research Center, 69120 Heidelberg, Germany; Federated Information Systems, German Cancer Research Center, 69120 Heidelberg, Germany; Faculty of Computer Science and Mathematics, Universität Passau, 94030 Passau, Germany; Department of Computer Science, Technische Universität Darmstadt, 64289 Darmstadt, Germany; Federated Information Systems, German Cancer Research Center, 69120 Heidelberg, Germany; Zentrum für Präventivmedizin und Digitale Gesundheit Baden-Württemberg, University Medical Centre Mannheim, 68135 Mannheim, Germany

## Abstract

**Motivation:**

Record Linkage has versatile applications in real-world data analysis contexts, where several datasets need to be linked on the record level in the absence of any exact identifier connecting related records. An example are medical databases of patients, spread across institutions, that have to be linked on personally identifiable entries like name, date of birth or ZIP code. At the same time, privacy laws may prohibit the exchange of this personally identifiable information (PII) across institutional boundaries, ruling out the outsourcing of the record linkage task to a trusted third party. We propose to employ privacy-preserving record linkage (PPRL) techniques that prevent, to various degrees, the leakage of PII while still allowing for the linkage of related records.

**Results:**

We develop a framework for fault-tolerant PPRL using secure multi-party computation with the medical record keeping software *Mainzelliste* as the data source. Our solution does not rely on any trusted third party and all PII is guaranteed to not leak under common cryptographic security assumptions. Benchmarks show the feasibility of our approach in realistic networking settings: linkage of a patient record against a database of 10 000 records can be done in 48 s over a heavily delayed (100 ms) network connection, or 3.9 s with a low-latency connection.

**Availability and implementation:**

The source code of the sMPC node is freely available on Github at https://github.com/medicalinformatics/SecureEpilinker subject to the AGPLv3 license. The source code of the modified Mainzelliste is available at https://github.com/medicalinformatics/MainzellisteSEL.

**Supplementary information:**

Supplementary data are available at *Bioinformatics* online.

## 1 Introduction

In medical research, many questions can only be addressed by combining data from different research institutions and clinics. New correlations between diseases and medical indications require combining data usually originating from different sources, such as genomic data, laboratory values or clinical data. In particular for rare diseases, individual treatment facilities will generally not have enough cases to be able to draw statistically significant conclusions. For this purpose, it is essential that data stored at different locations for a given patient are correctly linked (referred to as ‘record linkage’). At the same time, it is necessary to safeguard the patient’s privacy and manage the data according to applicable data protection laws (Eurpean Parliament and Council, 2016). In particular, personal patient data may not be stored or exchanged between different data sources across organizations without a sound legal basis, usually a patient’s informed consent (Vatsalan *et al.*, 2013).

Patient data stored in databases can be considered to consist of two components, namely (i**)** the identity data (IDAT, e.g. given name, surname, date of birth) and (ii**)** the medical data (MDAT). In this article, we will only be considering record linkage using information from the IDAT. One cannot always assume that patient data will be always complete and free of errors. Nonetheless, we would like to be able to match patient data across datasets wherever possible. In 1969, Fellegi and Sunter published the first mathematical description of record linkage, the process of pairwise comparison of records from two sets of records to find the pairs that likely represent identical entities (Fellegi and Sunter, 1969). The use of such record linkage methods, employing plaintext identifying data, is well established in the domain of patient data (Winkler, 2014). However, given that the two sets of patient data may be at geographically separated and legally independent institutions, it is clear that plaintext patient data will need to leave at least one of the sites for the comparison to be made, an obvious confidentiality issue.

To mitigate this problem, a number of techniques were developed, known as Privacy-Preserving Record Linkage (PPRL) (Brown *et al.*, 2017). These techniques transform the IDAT in such a way as to make the identification of the patient difficult, but still allow record linkage. One type of PPRL is based on ‘Bloom filters’ (Schnell *et al.*, 2009; Vatsalan *et al.*, 2013), which allow error-tolerant linkage of hashed identifying data. This approach has been implemented in the Mainzelliste, an open-source software (Lablans et al., 2015) used for pseudonymization and depseudonymization of patient IDAT and the administration of multiple pseudonyms. For reidentification of patients it uses a record linkage mechanism, based on the EpiLink algorithm (Contiero *et al.*, 2005), which allows for fault-tolerant patient matching. Mainzelliste is widely used in various medical research networks (Lablans et al., 2018; Miracum, 2019), patient registries (Burkhart and Wiese, 2015; Muscholl et al., 2014), a radiotherapy infrastructure for multicentric studies (Skripcak et al., 2016), centralized biobanks (Bernemann *et al.*, 2016) and commercial software (Climedo, 2019; iAS interActive Systems GmbH, 2019; Link, 2019).

However, even when using Bloom filters, identity data—albeit in encrypted form—is still being stored in a central location and can, in principle, be misused for unauthorized reidentification. In fact, many Bloom filter-based solutions are vulnerable to frequency and cryptanalysis attacks (Christen *et al.*, 2017; Vatsalan et al., 2017). Although a recent version is claimed to be secure against all currently known exploits (Schnell and Borgs, 2018), it can be expected, as with any computer system (Zabicki and Ellis, 2017), that new attacks could be devised to circumvent these improvements.

An optimal record linkage process would completely avoid storing IDAT—in any form—outside of the original treatment facilities and, thus, render such attacks impossible. Ideally, none of the record linkage parties would obtain any new information from the linkage process. This is the promise of Secure Multi-Party Computation (sMPC). This technique is based on the principle that in a computation performed across multiple parties, each participating party only knows their own input and the result of the given computation (Vatsalan *et al.*, 2013).

In this article, we describe the design and implementation of Mainzelliste Secure EpiLinker (MainSEL), a variant of sMPC integrated as an extension into Mainzelliste. A record linkage setup using MainSEL is comprised of a *local data source*, the *local MainSEL*, a *remote data source*, the *remote MainSEL* and (optionally) a *linkage service*. We developed a close integration into Mainzelliste, to deploy a holistic ID management and linkage solution based on an open-source software that is already in wide use.

### 1.1 Related work

While being studied for over fifty years, record linkage algorithms and techniques gained increased traction and interest in the last decade. In comparison to the classic publications of record linkage (Fellegi and Sunter, 1969) the focus shifted toward PPRL techniques to meet raising privacy requirements.

A number of techniques use Bloom filters and hash-based message authentication codes (HMACs) to provide privacy in the linkage process (Schnell *et al.*, 2009), which has been proven insecure, if additional security measures (e.g. usage of salts) are not taken (Kuzu *et al.*, 2011). Another active field of research is the scalability of PPRL methods (Vatsalan et al., 2017) or the incorporation of additional data types, like clinical and genomic data (Baker *et al.*, 2019). In the PPRL space, Laud and Pankova (2018) have recently leveraged sMPC to perform record linkage without a trusted third party for the iDASH 2017 competition. They used the Sharemind framework (Bogdanov *et al.*, 2008) to perform *exact-only* matching of databases.

#### 1.1.1 Comparison to current state-of-the-art

More recently, Lazrig *et al.* (2018) used sMPC and Bloom filter string comparisons with Dice-coefficients based on (Schnell *et al.*, 2009) to implement probabilistic PPRL. This is similar to our approach in that they use the same methodology for fault-tolerant string matching, since the Mainzelliste software’s record linkage algorithm also uses the method by Schnell *et al.* (2009). But our work differs to theirs in several ways.

They use a total of four Bloom filters, in which (fragments of) different fields are combined. Expert knowledge of probable errors is encoded in the choice of fragments and fields. In contrast, our solution takes a much more general approach by implementing the full field-tested EpiLink algorithm (Contiero *et al.*, 2005) as implemented in the Mainzelliste (Lablans *et al.*, 2015): fields are compared directly (and are not mixed up in the same Bloom filter), missing fields are handled properly and erroneously interchanged fields are handled by the introduction of *exchange groups* in the record linkage configuration.

Furthermore, our solution performs the whole post-processing of linkage information still in sMPC, whereas Lazrig et al.’s solution reveals per Bloom filter whether it matched, which leaks information (Revealing per-field matching information can leak, e.g. that someone has a different, say, surname in the other database, because the surname Bloom filter did *not* match. Another possible attack is to query the database multiple times, each time revealing a match for another field, like family name and DOB, thus iteratively re-identifying a record over multiple queries by intersecting on different fields. The real query stays invisible to the queried database. In general, if not the whole computation is performed in sMPC, unforeseen information leak can occur from intermediate values.). Our method also resolves ties between several probabilistically matching records by determining the highest match score before evaluating the score threshold. For this, we implemented a novel quotient-ordering circuit that is able to calculate the maximum of many quotients, with its index, in sMPC. Their solution cannot resolve ties between multiple matches. Additionally, our solution compares fields like the date of birth or zip code with exact equality and combines the individual field comparisons in a weighted sum to give the final score, before evaluating the score threshold, whereas, as mentioned above, their solution only performs a threshold comparison on individual field comparisons.

Hence our solution is more generally applicable and delivers a higher quality of matches (see Section 3.1 for the results and Supplementary Appendix SB for more details and a direct comparison to Lazrig *et al.*) and in particular contains Lazrig *et al.*’s method as a special simplistic case: Circuit 5 (Dice-coefficient) alone reflects their whole sMPC implementation (without the final threshold evaluation) while additionally being CBMC-GC-2 (Buescher *et al.*, 2016) optimized. They also built a custom implementation using Yao’s Garbled Circuit whereas we use the more general sMPC-framework ABY (Demmler *et al.*, 2015a) and offer four different protocol variants (cf. Section 2.3.3), so our solution can be more easily extended for future challenges regarding in-sMPC processing of match results. Also note that they did not publish their software whereas the source code of MainSEL is freely available under the AGPLv3 license. Furthermore, our solution can be deployed in hospital environments today as it is an extension of the already widely deployed Mainzelliste (Lablans *et al.*, 2015) patient record management solution and comes with a complete linkage service ID management solution.

Unlike Lazrig *et al.*, we choose to forego blocking mechanisms and instead compare all records, which leads to an extremely strong privacy guarantee, as many blocking techniques, especially techniques based on *Differential Privacy* (DP), are not composable with sMPC security guarantees. In fact, the security notion of DP is contrary to the sMPC security goals in the case of PPRL, as sMPC aims to reveal the correct, exact result of the ideal function and DP aims to reveal only noisy, i.e. (boundedly) approximate results. Even hybrid systems using differentially private blocking mechanisms as a pre-processing for the sMPC record linkage (Inan *et al.*, 2010), like Lazrig *et al.*, fail to achieve strong end-to-end privacy and may reveal properties of the inputs. The proof of those statements, as well as further complications at composing DP and sMPC techniques with a focus on PPRL are discussed in He *et al.* (2017).

That makes our work, to the best of our knowledge, the first practical sMPC-based probabilistic PPRL solution able to handle noisy and heterogeneous datasets which is also effectively handling incomplete data.

## 2 Materials and methods

We will describe the method and our implementation in great detail, giving hospitals, data protection commissioners as well as governmental regulators the details to evaluate this novel method.

In this article, the function log  denotes the logarithm to base 2. When we describe a protocol between two parties, we sometimes refer to them as Aarhus and Berlin (This is obviously inspired by the well known *Alice* and *Bob*, but changed to city names to stress the potential geographical distance of the protocol’s participants.) for increased clarity of the description.

### 2.1 Record linkage

Record linkage describes the task of linking records from different data sources that belong to the same entity. In general, this may include two or more data sources. We deal with the task of peer-to-peer, that is, two-party record linkage. In this setting, classic solutions off-load the record linkage to a *Trusted Third Party (TTP)*, often called a linkage unit. Since we use secure multi-party computation, we do not require any trusted third party.

Privacy-Preserving Record Linkage (PPRL) generally advances in several steps: data pre-processing, blocking/filtering, field comparisons/similarity and match classification. This is then followed by some application of the record linkage classification, e.g. the counting of matches (*match-cardinality*) or linkage of the datasets for scientific evaluations. The MainSEL software implements the field comparison, match classification and the match-cardinality application. In Section 2.4 we introduce a *linkage service* that enables the secure use of the linked datasets in arbitrary follow-up applications. Note that this service is not a TTP.

Given a record *x* and a set of *N* database records ${\left\{y^{j}\right\}}_{0\leq j<N}$ (abbreviated $\left\{y^{j}\right\}$), we want to determine the best matching database record and quantify the match quality. To that end, we will introduce a similarity score function *S*(*x*, *y*) between two records that attains values between 0 and 1. The database record with the highest similarity score is then compared to a threshold parameter (Mainzelliste actually checks against two thresholds, leading to a more fine-grained classification. We implemented this but omit its description for brevity.) 0<T≤1. Only if it is above this threshold, those records are identified as a match. We call this functionality $\text{bestMatch}(x,\{y^{j}\})$.

Let ${\left\{x^{k}\right\}}_{0\leq k<M}$ (abbreviated $\left\{x^{k}\right\}$) be a set of *M* records. For each *x^k^*, we determine the best matching record and check whether the score reaches the threshold. The count of those matches now determines the above introduced *match-cardinality*, which we denote with $\text{matchCardinality}(\{x^{k}\},\{y^{j}\})$.

In summary, we care about the following two functionalities:

(1) $$\begin{array}{c} \text{bestMatch}(x,\{y^{j}\}):=(j^{*},S(x,y^{j^{*}})>T)\\ \in \{0,\ldots ,N-1\}\times \{0,1\},j^{*}:=\underset{0\leq j<N}{\text{argmax}}(S(x,y^{j})) \end{array}$$

 

(2) $$\begin{array}{c} \text{matchCardinality}(\{x^{k}\},\{y^{j}\}):=|\{k:\exists j:S(x^{k},y^{j})>T\}|\\ \in \{0,\ldots ,\min(M,N)\} \end{array}$$

#### 2.1.1 Match classification score

To determine the similarity score of two records, we implemented the same algorithm as used by the Mainzelliste software, which is inspired by the EpiLink software (Contiero *et al.*, 2005) and resembles a threshold-based similarity join (Cohen, 2000). This leads to the best possible compatibility within the German medical research ecosystem, where the Mainzelliste is the most commonly used tool for data pseudonymization in medical research networks.

The similarity score *S*(*x*, *y*) of two records *x* and *y* is a normalized weighted sum of field similarities, yielding a score between 0 and 1:

(3) $$S(x,y):=\overset{s(x,y):=}{\overset{︷}{\sum_{i\in I}\delta_{i,i}w_{i}{\text{sim}}_{i}(x_{i},y_{i})}}/\overset{w(x,y):=}{\overset{︷}{\sum_{i\in I}\delta_{i,i}w_{i}}}.$$

The two records *x* and *y* have n=|I| field values *x_i_* and *y_i_*, each, for i∈I, where *I* is the field index set. $\delta_{i,j}$ is 1 if both fields *x_i_* and *y_j_* are non-empty and 0 otherwise. Similarity of fields of index *i* are determined using the functions ${\text{sim}}_{i}$, which will be described in Section 2.1.2. Following (Contiero *et al.*, 2005), the weights are chosen according to the formula $w_{i}=\text{log}((1-e_{i})/f_{i})$ where *e_i_* and *f_i_* are the error rate and average frequency of values, respectively. Those values are statistically derived once for a set of fields and then fixed, see Supplementary Appendix SD for the values we used.

Two fields are determined to match if their score is above a certain threshold. Weighting each field’s impact on the final score differently improves the score’s ability to veraciously categorize matches.

We introduced the definitions *s*(*x*, *y*) and *w*(*x*, *y*) for the numerator and denominator, which we also call the field-weight and weight component of a (partial) score, because we often need to work with them individually, especially when describing the sMPC solution. The actual division S=s/w is never evaluated.

*Tie-solving order*. We often need to determine the maximum of a set of quotients, for which we introduce a special order. On quotients $S_{1}=s_{1}/w_{1}$ and $S_{2}=s_{2}/w_{2}$, written as numerator-denominator pairs (*s*_1_, *w*_1_) and (*s*_2_, *w*_2_), we define the *tie-solving order* a*s* 
 

(4) $$\begin{array}{c} \left(s_{1},w_{1})>(s_{2},w_{2}\right)\\ :\Leftrightarrow (s_{1}w_{2}>s_{2}w_{1})\vee (s_{1}w_{2}=s_{2}w_{1}\wedge w_{1}>w_{2}), \end{array}$$

which returns true even if the quotients are the same, but numerator and denominator of the left quotient are nominally larger. This makes sense for our application because if the left field-weight/weight quotient is nominally larger, then more entries contributed to its score (i.e. more entries of the right quotient were empty). It also solves the problem of zero denominators, favoring the quotient with non-zero denominator in such a case. If both are zero, it does not matter which one is chosen by this order, as the contribution to a sum would then be zero anyway.

*Exchange groups*. In real record linkage scenarios, linkage quality can be improved by grouping some fields into so-called *exchange groups*, like *first, sur-* and *birth name*. Such fields may be accidentally swapped when entered. The score (3) is now modified to pairwise compare all fields of an exchange group.

Let Sym(G) denote the set of all permutations of a set *G*, its *symmetric group*. It has size |G|!. We introduce the following useful definitions of the sum of weighted similarity scores and sum of weights for an exchange group G⊂I and permutation σ∈Sym(G):

(5) $$\begin{array}{c} s_{G}^{\sigma }:=\sum_{i\in G}\delta_{i,\sigma (i)}w_{i,\sigma (i)}{\text{sim}}_{i}(x_{i},y_{\sigma (i)}),\\ w_{G}^{\sigma }:=\sum_{i\in G}\delta_{i,\sigma (i)}w_{i,\sigma (i)},\\ w_{i,\sigma (i)}:=\frac{w_{i}+w_{j}}{2}. \end{array}$$

A group *G*’s sub-score for permutation *σ* is now defined as $S_{G}^{\sigma }(x,y):=s_{G}^{\sigma }/w_{G}^{\sigma }$. Note that all fields in *G* must be of the same comparison type and that $S=S_{I}^{\text{id}}$. The score *S_G_* for a group *G* is now determined as the maximum of all sub-scores for all permutations, using the tie-solving order (4):

(6) $$S_{G}(x,y)=(s_{G},w_{G}):=\underset{\sigma \in \text{Sym}(G)}{\max}(s_{G}^{\sigma },w_{G}^{\sigma }).$$

Let E  be the set of all exchange groups and $\tilde{I}\;:=I\setminus \cup_{G\in E\;}G$ those fields not in any exchange group. Our final similarity score of two records *x* and *y* now become*s* 
 

(7) $$\begin{array}{c} S(x,y)=s(x,y)/w(x,y)\\ =(\sum_{G\in E\;}s_{G}+s_{\tilde{I}\;}^{\text{id}})/(\sum_{G\in E\;}w_{G}+w_{\tilde{I}\;}^{\text{id}}), \end{array}$$

where *s_G_* and *w_G_* are the numerator and denominator of the group scores *S_G_*, as defined in Eq. (6).

#### 2.1.2 Field comparison

Depending on the field type of field *i*, we use either simple equality or Dice-coefficients of Bloom filters (Bloom, 1970) (introduced below) as the measure of similarity ${\text{sim}}_{i}$. Equality comparison is applied to numeric and other data fields where no matching fault tolerance is wanted. It simply assigns 1 to fields that are exactly the same and 0 otherwise.

*Bloom filter dice similarity*. The Bloom filter Dice similarity from Schnell *et al.* (2009) is applied to string fields like *first* and *surname* where fault tolerance is desired. Strings *x* are converted into a Bloom filter Bl (x) by tokenizing them into bigrams and then applying a family of hash functions to the bigrams, thereby setting the bits in the Bloom filter bitmask. Further details can be found in Supplementary Appendix SA.

Let Hw denote the *Hamming-weight*, that is, the number of set bits of a bit vector and X∧Y denote bitwise AND of the bit vectors *X* and *Y*. Write $H_{x}:=\text{Hw}\;(\text{Bl}\;(x))$ for the Hamming-weight of the Bloom filter of string *x* and $H_{x\wedge y}:=\text{Hw}\;(\text{Bl}\;(x)\wedge \text{Bl}\;(y))$. Using the *Sørensen-Dice*-coefficient (Dice, 1945), the similarity of two strings is now calculated a*s* 
 

(8) $${\text{sim}}_{\text{string}}(x,y)=\frac{2\cdot \text{Hw}\;(\text{Bl}\;(x)\wedge \text{Bl}\;(y))}{\text{Hw}\;(\text{Bl}\;(x))+\text{Hw}\;(\text{Bl}\;(y))}=\frac{2\cdot H_{x\wedge y}}{H_{x}+H_{y}}.$$

**Fig. 1.**
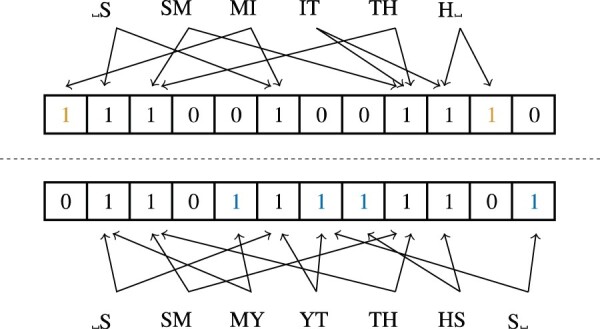
Visual example of a Bloom filter-based Dice similarity measurement between the strings ‘SMITH’ and ‘SMYTHS’. Differences in the set bits are colored. This example assumes *k *=* *2 independent hash functions and a 12 bit Bloom filter. Note that a change of one letter leads to at most 2*k* changes in the Bloom filter. This means that small changes in the strings lead to small changes in the bit vector

The Dice-coefficient has the advantage of being insensitive to the number of zero bits, of which there will be many for a large Bloom filter. It captures the *relative* similarity of strings. An example is shown in Figure 1. Note that the Dice-coefficient could also have been applied directly to the bigrams of two strings in a similar way. Unlike many other PPRL algorithms, we do not rely on Bloom filters for increased privacy of the input data (In fact, it has been shown insecure by Christen *et al.* (2017)). For privacy, we rely on sMPC instead, and Bloom filters are only used because they can be evaluated more easily in sMPC than bigrams.

### 2.2 Secure multi-party computation

The central component of our system is based on a technique called *secure multi-party computation (sMPC)*. While the foundations of this subfield of cryptography were laid in the 1980s (Goldreich *et al.*, 1987; Yao, 1986), it has long been considered impractical due to the large computational overhead. This changed in the early 2000s, with cryptographic breakthroughs such as Oblivious Transfer Extensions (Ishai *et al.*, 2003) and the first implementation of generic two-party computation (Malkhi *et al.*, 2004). Since then, a variety of sMPC frameworks have been developed (Bogdanov *et al.*, 2008; Damgrd *et al.*, 2012; Demmler *et al.*, 2015a; Zahur and Evans, 2015). In this work, we rely on the ABY framework (Demmler *et al.*, 2015a) for secure two-party computation.

ABY implements three approaches to two-party computations: Yao’s Garbled Circuit (Yao, 1986), the GMW protocol (Goldreich *et al.*, 1987) and Arithmetic Sharing, as well as transformations of intermediate values between them. These protocols and their security assumptions are outlined in Supplementary Appendix SC.

This allows us to freely combine those techniques, as some operations are more efficient in a specific sMPC protocol. In Section 2.3, we will present the details of our circuit designs and in Section 3.4 we explore how different combinations of sMPC protocols affect the running time of our implementation.

#### 2.2.1 Threat model and privacy goals

Ideally, we would like to guarantee that in our system, no information about the input data can be learned by anyone at all. However, this definition of privacy is not very useful, since the output of any meaningful computation necessarily contains information about the inputs. Therefore, the cryptographic definition of privacy for sMPC protocols draws an analogy to a Trusted Third Party (TTP): Informally, a distributed protocol is said to *privately implement* an *ideal functionality f*, if the information revealed by the protocol is the same as what would be revealed if a TTP had computed *f*. We omit a formal definition here and instead refer to Goldreich (2004). The ideal functionalities considered in this work are bestMatch and matchCardinality from Eqs. (1) and (2).

Throughout this article, we will focus on the *semi-honest* or *honest-but-curious* attacker model. In this setting, protocols aim to be secure against an attacker who correctly follows the protocol, but additionally tries to learn as much as possible about the other parties’ inputs and outputs. While stronger security models (such as *covert* or *malicious* adversaries) exist, the semi-honest model is a good fit in a setting as ours, where the parties are regulated by law and known in advance.

While the inputs of the parties participating in an sMPC protocol remain secure, the input *sizes* (i.e. the number of records to be linked) need to be known in advance. However, in cases where this information is still considered sensitive, the parties can pad their databases with dummy elements to any reasonable upper bound on the size, thus hiding the actual size, at the expense of increased computational complexity.

Summing up ABY’s security assumptions (cf. SC.3), the weakest link in the security of our ABY implementation is, apart from supporting only the semi-honest attacker model, the reliance on the quantum-insecure CDH assumption. Accepting this assumption, our implementation guarantees that each party of the sMPC does not learn anything about the other party’s input or any intermediate calculation. They only learn what is specified as the ideal functionality’s output: in the single-record-linkage mode of operation (bestMatch), they each learn one part of an XOR sharing of the matching record’s index together with the match bit. This is then forwarded to the linkage service as described in Section 2.4 for further processing. Each XOR share looks just like random data to each party. In the match-cardinality mode of operation (matchCardinality), both parties only learn the number of matches.

### 2.3 Circuit design

In order to calculate the main functionalities (1) and (2) in a sMPC with ABY, they have to be expressed as circuits. The following sections give an in-depth description of the circuit designs of the score calculation (7) and how to determine the maximum of those scores, which leads to bestMatch  (1). We choose to work in fixed-point arithmetic because it is more efficient for our purpose, although floating-point calculations are partially possible in ABY (Demmler *et al.*, 2015b).

When expressing algorithms in a circuit, it cannot have dynamic control flow, because otherwise information of intermediary results would leak. Thus, all branches have to be evaluated and all loops have to be unrolled. For efficiency reasons, all unrolled loops are executed in parallel and all sums are calculated as balanced binary-trees to minimize the circuit depth.

Because we lack dynamic control flow, we decided to not apply blocking mechanisms, as is usually done in record linkage pipelines. Blocking describes the pre-filtering of records such that less records need to be fully compared. We also have to evaluate all field similarities, even if either field is empty (in which case this pair of fields does not contribute to the score).

Given a record *x* by Aarhus and the records $\left\{y^{j}\right\}$ by Berlin, the circuit

C 1. calculates all scores’ numerators $s(x,y^{j})$ and denominators $w(x,y^{j})$  *(*cf. eq. (7)),

C 2. determines the highest score and its index $j^{*}:=\text{arg}{\text{max}}_{j}S(x,y^{j})$,

C 3. tests for a match by calculating the *match bit*

 

$$\{\begin{array}{cc} 1, & \text{if}\;S(x,y^{j^{*}})=s(x,y^{j^{*}})/w(x,y^{j^{*}})>T\\ & \;\;\;\;\;\;\;\;\;\;\;\;\;\;\;\;\;\Leftrightarrow s(x,y^{j^{*}})>Tw(x,y^{j^{*}}),\\ 0, & \text{otherwise}. \end{array}$$

It is more efficient to calculate the field-weight- and weight-sums *s* and *w* in parallel, use them for (C2) and (C3) and never actually calculate the divisions S=s/w. The steps (C1)–(C3) combined completely implement the functionality $\text{bestMatch}(x^{k},\{y^{j}\})$. If Aarhus now has *M* records $\left\{x^{k}\right\}$ and they want to compute the $\text{matchCardinality}(\{x^{k}\},\{y^{j}\})$, they compute $\text{bestMatch}(x^{k},\{y^{j}\})$ for all *k* and then simply sum the match bits.

#### 2.3.1 Notation

To describe the individual circuit components, we introduce the notation x=C(x) to say that x is the encoding of value *x* as a circuit input or the circuit implementation of function *x*. Sans-serif font is used for circuit variables, typewriter for circuit functions/algorithms. We define bitlen(x):=bitlen(C(x)):=bitlen(x):=l, for $x\in {\left\{0,1\right\}}^{l}$ or $x:*\to {\left\{0,1\right\}}^{l}$. We sometimes abbreviate the three sMPC protocols Arithmetic Sharing, GMW and Yao with *A*, *B* and *Y* (This adheres to the notation of ABY.), respectively, and denote the spaces of values of bit-length *l* in those protocols as $S_{A}^{l},S_{B}^{l}$ and $S_{Y}^{l}$. We also introduce the annotation ${\langle x\rangle }_{p}^{l}$ of a variable’s or function’s output bit-length *l* and protocol p=A,B or *Y*. It is mainly relevant to the discussion in Section 2.3.5. The superscript bit-length *l* or subscript protocol *p* are sometimes omitted for brevity.

#### 2.3.2 Fixed-point representation

We start by introducing the fixed-point presentations of weights and field similarities, which are the only two real number variables in the similarity score. Let $\mathrm{lw}:=\mathrm{bitlen}(w_{i})$ be the *weight precision* and $\mathrm{ls}:=\mathrm{bitlen}({\text{sim}}_{i})$ the *similarity* or *Dice precision*, which is the same for all fields i∈I.

The field similarity measures ${\text{sim}}_{i}$ output real numbers between 0 and 1. So their fixed-point representations are calculated as $C({\text{sim}}_{i})=\lfloor {\text{sim}}_{i}\cdot 2^{\mathrm{ls}}\rceil$. In case of equality, which outputs either 0 or 1, this is just a left-shift by ls. The circuit implementation of the Bloom filter Dice-coefficient ${\text{sim}}_{\text{string}}$ uses a custom integer-division where the numerator is left-shifted by ls before the integer-division to give a result between 0 and $2^{\mathrm{ls}}$.

Similarly, the real-valued threshold *T* is multiplied with $2^{\mathrm{ls}}$ and rounded to the nearest integer to attain its fixed-point representation, $T=C(T)=\lfloor T\cdot 2^{\mathrm{ls}}\rceil$. It is necessary to scale the threshold together with the field similarities to make inequality (C3) work.

The real weights $w_{i}>0$ are transformed into numbers $w_{i}=C(w_{i})\in {\left\{0,1\right\}}^{\mathrm{lw}}$ by rescaling them so that the highest weight has value $2^{\mathrm{lw}}-1$ and then rounding to the nearest integer:

(9) $$w_{i}:=\lfloor \frac{w_{i}}{w_{\text{max}}}(2^{\mathrm{lw}}-1)\rceil ,\;w_{\text{max}}:=\underset{i\in I}{\max}w_{i}.$$

This leads to the highest possible precision because the weights occupy the full range of ${\left\{0,1\right\}}^{\mathrm{lw}}$.

#### 2.3.3 Implementation variations

As has been shown (Demmler *et al.*, 2015a), using different protocols for different kinds of calculations with intermediate conversions may be more efficient than staying in the same protocol, even if this incurs additional conversion costs. We therefore implemented four variations of the circuit, choosing different sMPC protocols for Boolean/logic (protocol β) and arithmetic (protocol α) components of the circuit, with possible conversions in between where necessary. Points of possible conversions are denoted with α2β and β2α. Note that they are *no operation* if the same protocol is chosen for α and β.

For the Boolean/logic components, either the GMW or Yao’s Garbled Circuit protocol were selected, i.e. β=B or *Y*. Additionally, for the arithmetic circuit components, we either stayed in the same protocol β, or converted to Arithmetic Sharing, i.e. α=β or *A*. This results in the following four circuit variants.

GMW : β=α=B, i.e. the whole circuit implemented in the GMW protocol.

GMW/A : β=B and α=A, i.e. Boolean/logic components implemented in the GMW protocol and arithmetic components in Arithmetic Sharing.

Yao : β=α=Y, i.e. the whole circuit implemented in Yao’s Garbled Circuit.

Yao/A : β=Y and α=A, i.e. Boolean/logic components implemented in Yao’s Garbled Circuit and arithmetic components in Arithmetic Sharing.

Specifically, Circuits 2 and 3 are of arithmetic nature while Circuits 4 and 5 are of Boolean nature. Circuit 6 is of mixed nature: after two multiplications, several Boolean operations are performed.

#### 2.3.4 Circuit components

We now describe the circuit implementations to attain results (C1)–(C3). Inputs are only mentioned in the circuit sub-component where they are used, thus omitted in superordinate components. Private inputs by Aarhus and Berlin are stated as semicolon-delimited pairs. A high-level overview of the circuit layout is shown with Circuit 1.

Now follows the description of the circuit implementation of Score with its sub-components for a single pair of records $x={\left\{x_{i}\right\}}_{i\in I}=C(x)$ and $y={\left\{y_{i}\right\}}_{i\in I}=C(y)$ as private inputs by Aarhus and Berlin, respectively. For brevity, we omit the record index *j* for Berlin ’s input $y^{j}$ as only a single pair of records is relevant in the remaining section. Remember that $z_{i}$ denotes an *individual field* of a single record z.

Circuit 2 (Score) calculates the score numerators s=C(s) and denominators w=C(w) in parallel, in protocol α [cf. Eq. (7)]. It uses the sub-components GroupFieldWeight (Circuit 3) for the calculation of a group’s sub-score and MaxQuotient to determine the score for each group, i.e. the maximum over all group sub-scores [cf. Eq. (6)].

**Circuit 2:**
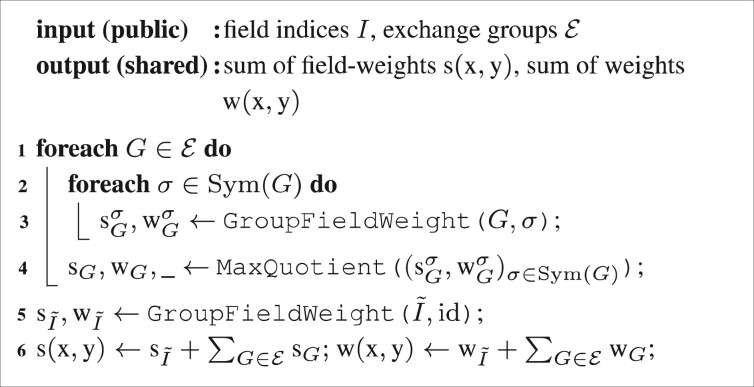
Score : similarity score (C1) of x and y [Eq. (7) in protocol α].

**Circuit 3:**
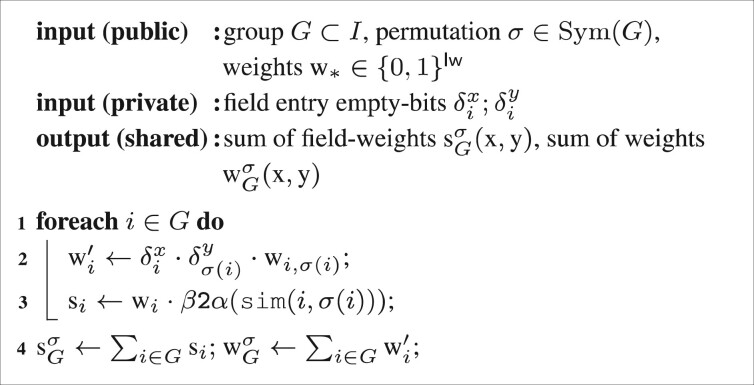
GroupFieldWeight [Eq. (5) in protocol α]. The empty-bits $\delta_{i}^{z}$ are 0 if entry *i* of record *z* is empty and 1 otherwise. If any entry is empty, then $w\prime_{i}=s_{i}=0$. Note that if α=B or *Y*, the multiplication between the *δ*’s in line 2 is a logical AND.

*Similarity circuits*. The field similarity sim applies either the simple equality Circuit 4 or the Bloom filter Dice-coefficient Circuit 5 on field entries $x_{i},y_{\sigma (i)}$, depending on their type. If field *i* has Dice similarity type, we use the field entry’s Bloom filter as the input to the circuit, $x_{i}=C(x_{i})=\text{Bl}\;(x_{i})$, which can be computed locally. The bit-length of field *i* is denoted by $lb_{i}$.

**Circuit 4:**
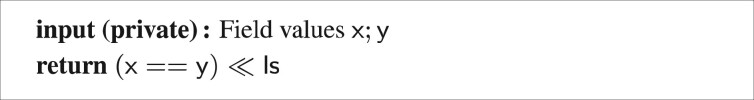
equal (in protocol β).

Note that both circuits output the similarity as values in protocol β of fixed-point precision ls. Free bit-shifts were used for multiplication or division by 2. The marked component of the dice circuit was created using the CBMC-GC-2 compiler (Buescher *et al.*, 2016; Franz *et al.*, 2014) on the function x,y↦((x≪ls+y/2)/y), which is rounding integer division (‘/’ and IntDiv denotes C integer division). A separate circuit was compiled for all feasible input and output bit-lengths 2≤lh+1≤12 and 2≤ls≤22=⌈64/3⌉, covering Bloom filters of length up to 2047. The Hamming-weight of a Bloom filter z needs lh=⌈ log(lb+1)⌉ bits, being the sum of lb many 1-bit numbers.

*Maximum quotient circuit*. Remember that a group’s sub-score is the quotient $s_{G}^{\sigma }/w_{G}^{\sigma }$. As described in Section 2.1.1, a group’s score is the maximum of those sub-scores [cf. Eq. (6)] and is determined by evaluating a fold with the tie-solving order as defined in eq. (4). This order is implemented in Circuit 6, which outputs the larger of two quotients, together with its index.

**Circuit 6:**
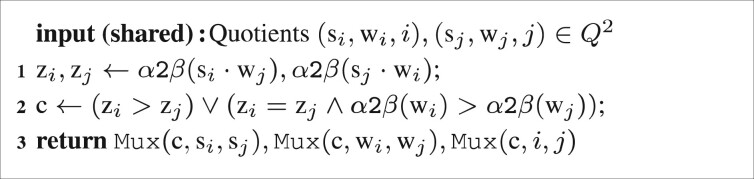
$\mathrm{MaxQuotient}\prime :Q^{2}\to Q$ (maximum of two quotients with index in mixed protocols). It is $Q:=S_{\alpha }\times S_{\alpha }\times S_{\beta }$ for the space of quotients together with their indices and Mux(c,a,b) returns a if c is 1 and b otherwise (c∈{0,1}).

Note that the index is not needed for the calculation of a group weight, but will later be used when calculating the maximum over all scores to determine the best match in Circuit 1.

Now the actual MaxQuotient circuit is the binary-tree fold of a list of quotients using MaxQuotient′ as the fold operation.

#### 2.3.5 Precision choices and overflow prevention

It follows a discussion about the chosen bit-length *L* for arithmetic circuit components and the resulting fixed-point precisions lw and ls to prevent overflows. The weight sum w(x,y) of Circuit 2 is a sum of *n* weights of bit-length lw and as such has length ⌈ log(n)⌉+lw. Similarly s(x,y) has length ⌈ log(n)⌉+lw+ls. However, the largest values that are created in any arithmetic circuit component are the $z_{i}$’s of Circuit 6, line 1. Multiplying s with w means multiplying a sum of *n* weights of length lw with a sum of *n* field-weights of length lw+ls, resulting in a variable of bit-length $\left\lceil \;\text{log}(n^{2})\right\rceil +2\mathrm{lw}+\mathrm{ls}$. Hence lw and ls are chosen such that the bit-length *L* of space $S_{\alpha }^{L}$ is fully used but no overflows occur (ABY supports L∈{8,16,32,64} if α= Arithmetic Sharing): we have $r:=L-\left\lceil \;\text{log}(n^{2})\right\rceil$ bits left to distribute to lw and ls. To distribute them evenly and, at the same time, not waste a bit, we set lw=⌈r/3⌉,ls=⌊r/3⌋ if r mod 3=2 and lw=⌊r/3⌋,ls=⌈r/3⌉ otherwise.

We compared the fixed-point score calculation as implemented in our circuits to the same calculation done in double floating point precision on a large number of random inputs. The observed deviations are < 1% for L=16 bit, < 0.1% for L=32 bit and negligible for L=64 bit. Most reported benchmarks in Section 3 were performed with *L *=* *32 and *n *=* *8 fields, such that lw=9 and ls=8.

**Circuit 1:**
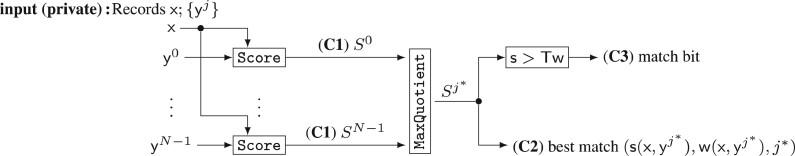
High-level circuit calculating (C1)–(C3), thus implementing functionality bestMatch. The scores $S^{j}:=S(x,y^{j})$  *(*result (C1)) are calculated by running Circuit 2 (Score) for all record input pairs x and $y^{j}$ from Aarhus and Berlin, respectively, in parallel. The best match (C2) is then determined by running circuit MaxQuotient on all scores, which is a balanced binary-tree fold of Circuit 6. Finally, the match bit (C3) is determined by evaluating inequality $s(x,y^{j^{*}})>\mathrm{Tw}(x,y^{j^{*}})$ on the best match.

**Circuit 5:**
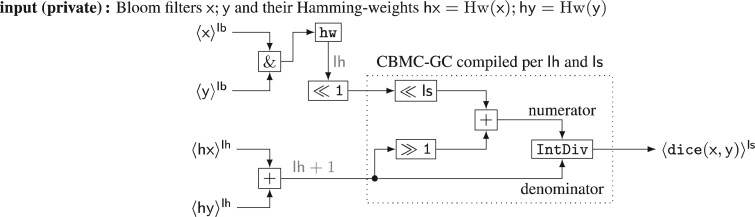
dice [Eq. (8) in protocol β] with annotated bit-lengths.

### 2.4 Systems architecture

In this section we describe the MainSEL record linkage system’s design. It is comprised of the Mainzelliste as the data source and SEL as the sMPC compute unit. Both components communicate with each other via JSON REST interfaces. We illustrate the systems’ communication interface, the record linkage and ID management workflow and possible additional modes of operation.

#### 2.4.1 Communication

The sequence of communication is divided into two phases: the initialization phase and the linkage phase. During local initialization the connection to the local data source and the structure of the records, as well as their weights, are configured. Then an arbitrary number of remote targets can be configured. Every call between each of the parties is authenticated via a pre-shared key and executed over a secure channel, e.g. TLS-secured (Rescorla, 2008).

The initialization phase is completed when the configurations between the local SEL and the (multiple) remote SEL s, as well as between the local SEL and the linkage service, are tested. The test assures connectivity and compatible algorithm configurations.

**Fig. 2.**
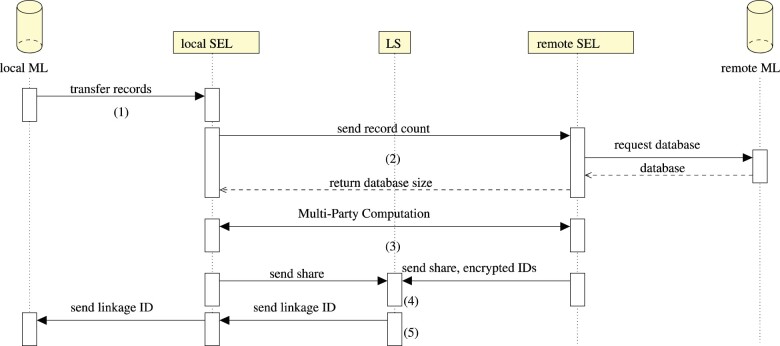
Communication sequence diagram of the linkage phase. *ML* stands for Mainzelliste, the patient database and pseudonymization framework, *SEL* stands for the sMPC compute unit and *LS* stands for Linkage Service. The communication proceeds over a secure, authenticated channel. The numbers in parentheses enumerate the protocol’s steps described in Section 2.4.1.

The linkage phase (see Fig. 2) starts with the local Mainzelliste sending one or a number of records to the local SEL and a callback address for the linkage result [step (1)]. The number of records to link, as well as the number of records in the remote Mainzelliste, need to be known for circuit creation. Therefore, the local SEL transmits its number of records to the remote SEL, which in turn queries all records from its remote Mainzelliste and returns that number [step (2)]. To allow a separation of circuit generation and linkage procedure, both numbers can be based on estimates and padded to allow growth in the time between circuit generation and linkage. In this case, it must be verified that the sizes used during the circuit generation are compatible with the actual numbers.

With these requirements satisfied, the actual sMPC is executed between the local and the remote SEL [step (3)]. At the end of the computation, each side holds one share of the index of the best match, as well as shares of the match bits. These shares are then sent to the linkage service. Additionally, the remote SEL sends their encrypted IDs to the linkage service [step (4)]. It combines the shares and the best matching IDs are de- and re-encrypted. The information whether a match occurred is stored together with the linkage ID (LID) in encrypted form. This LID is transmitted to the local SEL, which sends it to the given callback address for storage or evaluation in the Mainzelliste [step (5)].

#### 2.4.2 ID generation and management

The usage of the record linkage process results is a privacy concern in itself. To avoid re-identification by two colluding actors on both sides, the returned LID must not reveal any information about the matching result. However, this very information is the basis for the detection of duplicates and the assignment of pseudonyms.

Confidential pseudonymization is achieved by introducing the *Linkage Service*, a component only concerned with generating and encrypting LIDs. This component does not constitute a trusted third party, as it has no functionality in the linkage process and does never receive any private information. It only holds a secret key for every party for re-keying the generated LIDs and generates random IDs. This setup is used to prevent collusion of adversaries in both locations.

To prepare for confidential LID management, one data source contacts the Linkage service to generate random IDs for all its records. Those random IDs are encrypted with the corresponding party’s secret key. After receiving the linkage result as well as the list of IDs from the server, the linkage service decrypts the LIDs. If the linkage results in a match, a matching bit is concatenated to the decrypted ID and this string is re-encrypted with the client’s secret key. If the records do not match, a new random ID is generated and encrypted with the client key. This procedure ensures that every LID looks like a random string and even two actors on both sides are not able to examine or compare IDs to learn matching records.

To identify the matching records, a process that requires the patients’ consent for the data exchange from both parties is executed which grants the linkage service permission to decrypt the LIDs and distribute them in plain text. This information allows the identification of matches as well as the quality of matches.

In this scenario, only the linkage service is allowed to generate LIDs. Otherwise the security of this procedure would be compromised. To check the validity of signatures, MainSEL uses a randomly chosen but sufficiently long zero padding of the plain text LIDs. This enables the linkage service to verify the validity of LIDs and that they belong to the correct party.

*LID Generation without a LS*. The described outsourcing of ID management is desirable for regulatory reasons, but not required from a cryptographic protocol perspective. The same functionality could be realized within the sMPC circuit, for example in the following way: both parties input an additional randomness per record. If bestMatch determines a match, both parties’ randomness is XORed to obtain the LID for both. Otherwise, each party just receives the other party’s randomness as LID. Both cases are without collusion indistinguishable, as in the matching case the LID is effectively One-Time-Pad-encrypted with the other party’s randomness and as such indistinguishable from a random string as in the second case. Only after direct comparison of the LIDs can actual matches be identified. The linkage service prohibits exactly this collusion and allows a structured process, like distributing the LIDs after a review procedure.

#### 2.4.3 Match-cardinality mode

As described in the introduction of Section 2.1, we can easily extent the bestMatch functionality to count the number of matches, resulting in functionality matchCardinality, by simply summing the match bits. This can also be interpreted as the (fault-tolerant) patient lists’ intersection cardinality.

This mode of operation is relevant for a number of real world applications, especially in the research and treatment planning of rare diseases. Patients with rare diseases are regularly recorded in multiple hospitals and research facilities, often with differing or uncertain diagnoses. This leads to a high amount of duplicate records in joint cohort studies. The current legal process for finding those duplicates includes all legal requirements required for transferring and processing the complete identifying dataset. This process is unreasonably complex for the feasibility analysis stage of a study, where e.g. cohort sizes are determined.

## 3 Results

This section provides benchmarking results for our implementation of the sMPC circuit as set forth in Section 2.3 and describes the experimental setup. The interpretation of the reported benchmarks is discussed in Section 4.

### 3.1 Record linkage quality

As we implement the established, well understood record linkage algorithm of the Mainzelliste software (Lablans *et al.*, 2015) that is in broad practical use in the German medical research environment, the analysis of the achieved record linkage quality is not the focus of this work. However, we can report a precision of 0.994 and perfect recall performing a linkage between two datasets with 10 000 (synthetic) records, each, 60% overlap and a 10% error rate per field. For more details on the data generation and perturbation procedure, as well as more analysis results and a comparison to Lazrig *et al.* (2018), we refer to Supplementary Appendix SB.

### 3.2 Benchmarking setup

For the implementation, we used C++ and the ABY framework (Demmler *et al.*, 2015a). The timing benchmarks ran on two identical servers with Intel Xeon E5-2690 CPUs (2.90 GHz), 256 GiB RAM each and a local 1 Gbit/s connection. Both ran a recent Arch Linux OS with vanilla Kernel version 4.20.7 and gcc version 8.2.1 for source code compilation. In ABY, we set the security parameters to achieve a symmetric security level of 128 bit. We furthermore chose L=32 bit as the bit-length of the arithmetic circuit components to achieve a score accuracy within 0.1%, cf. Section 2.3.5. All reported timings are averaged over at least five iterations. All benchmarks—except where specifically noted—are using the default EpiLink configuration that is shipped with the Mainzelliste software (Supplementary Table S6 in Supplementary Appendix SD), consisting of four Dice-compared and four equality-compared fields. The parameters of this default configuration are chosen following Sariyar *et al.* (2011).

### 3.3 Setup and online phases

Since a sMPC computation can be split into two phases, we report those timings separately. In the first *setup* phase (often called *offline* phase in the sMPC literature), only the size and structure of the circuit need to be known, but the input data can be set later. More specifically, in this phase the parties perform base OTs and OT-extension and exchange multiplication triples (Arithmetic Sharing) or Yao keys. Details can be found in the description of the ABY framework (Demmler *et al.*, 2015a). This allows for the—usually much more communication intensive—setup phase to be run before the input to the circuit is even known. The second *online* phase runs once the input to the circuit is known and usually requires an order of magnitude less communication and thus runs much faster than the setup phase.

Our record linkage circuit only depends on the database size and the EpiLink fields configuration, which is assumed not to change once two institutions agreed on a common configuration. Thus, two institutions running the Secure EpiLinker can greatly benefit from this separation into setup and online phase. They can run the setup phase on their combined databases, and once one side inserts a new patient in their database, they can immediately execute the online phase. We therefore often speak of the online runtime as the actual runtime of a secure record linkage procedure. To be fair, however, in an initial full database cross-linkage procedure, both phases’ timings would sum up to give the total runtime. On the other hand, the full cross-linkage would only need to run once after two institutions agree to enter the mutual secure record linkage scheme.

### 3.4 Timings

**Fig. 3.**
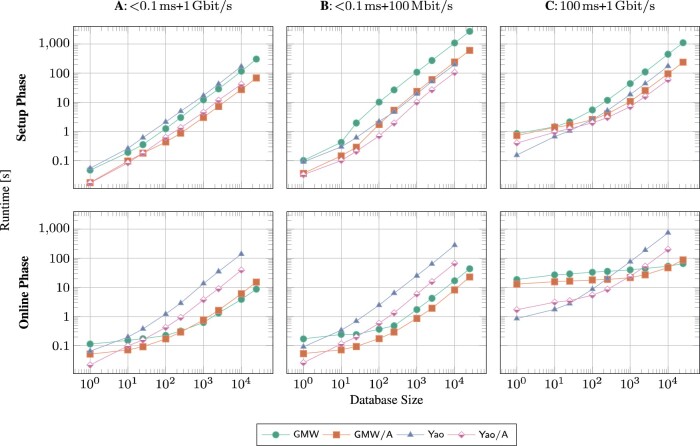
Setup and online runtime in seconds for *varying database sizes* and four circuit variants (cf. Section 2.3.3), in three network environments: (**A**) <0.1 ms latency  + 1 Gbit/s bandwidth, (**B**) <0.1 ms + 100 Mbit/s, (**C**) 100 ms + 1 Gbit/s. The Epilink configuration of DKFZ’s Mainzelliste (Supplementary Table S6 in Supplementary Appendix SD) was used in all benchmarks

Figure 3 reports the two phases’ runtimes for varying database sizes and sMPC circuit implementations, in three different network environments. We varied the database sizes from 1 to 10 000 and tested all four variants of the circuit implementation described in Section 2.3.3.

The pure Yao protocol has a constant number of rounds. The communication rounds of the other protocols are proportional to the circuit depth. Table 1 reveals that the number of communication rounds grows logarithmically with the database size, starting with an offset. For example GMW/A requires 266 rounds for database size one, reaching 506 for size 25 000. This can be explained by the fact that the first part of any circuit runs the record linkage for all database entries in parallel, resulting in a circuit of fixed depth not dependent on the database size. The second part of the circuit determines the maximum score in a balanced binary-tree, which explains the logarithmic growth.

**Table 1.** Comparison of the setup and online runtimes of the sMPC linkage procedure of a single record with a remote database in circuit variant GMW/A

Database	Comm. [MiB]			Setup phase [s]			Online phase [s]		
Size	No. of rounds	Setup	Online	A	B	C	A	B	C
1	266	0.6	0.1	0.018	0.036	0.72	0.052	0.054	13
10	330	5.5	0.7	0.097	0.15	1.4	0.072	0.072	16
25	346	13.5	1.7	0.18	0.29	1.6	0.093	0.094	17
100	378	53.7	6.7	0.43	1.7	2.5	0.17	0.17	18
250	394	133.9	16.8	0.87	5.3	4	0.29	0.3	19
1000	426	555.2	47.1	3	23	11	0.77	0.87	22
2500	458	1394.1	119.5	7.3	60	25	1.6	1.9	27
10 000	490	5577.4	459.4	28	240	96	6.1	8.2	48
25 000	506	13 917.9	1150.3	69	610	240	15	23	88

*Note*: Compared are the three networking configurations from Figure 3, for *varying database sizes*. The reported network communication cost is the sum of sent and received data. See Appendix SE for the complete set of tables.

Overall, the GMW/A variant performs best in both, the setup and online phase and almost all network settings. For all circuit variants, asymptotically, the sMPC runtime grows linearly, after a ramp-up for small database sizes. The ramp-up is more pronounced for non-Yao based variants, which can be explained by the previously discussed effect on the communication rounds. This is particularly visible for the online phase in network environment **C**. For larger database sizes, the larger amounts of data per round amortize the negative effects of multiple rounds and the bandwidth becomes the dominant effect on runtime.

**Fig. 4.**
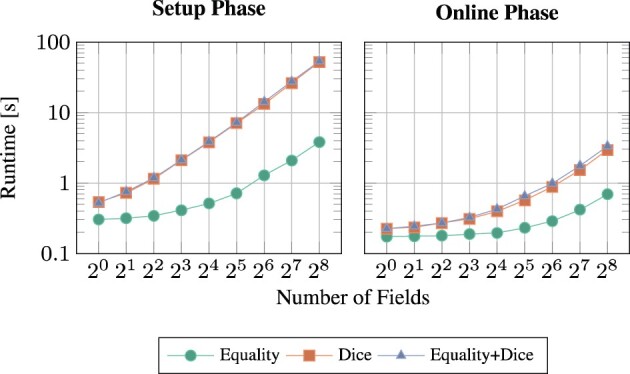
Setup and online runtime in seconds for *varying number of fields* and *varying field types*: (1) only 12 bit integer fields with equality comparison, (2) only 500 bit Bloom filters with Dice comparison or (3) both, counted as pairs. Network environment A: <0.1 ms latency + 1 Gbit/s bandwidth was used with a database size of 1000 and the GMW/A circuit variant

In Figure 4, a similar pattern can be seen for a growing number of fields (where the database size was kept constant at 1000). Asymptotically, the runtime grows linearly with the number of fields. This can be explained by the same arguments as before, because multiple fields are also compared in parallel. Also note that the runtimes of equality-compared integer fields are almost negligible in comparison to Dice-compared Bloom filter fields, because the latter are much more complex to evaluate (cf. Circuits 4 versus 5).

## 4 Discussion

In circuit variant GMW/A and network environment **A** (no latency, 1 Gbit/s), a full cross-linkage of two medium-sized databases with 10 000 patients each would take 78 h for the setup and 17 h for the online phase, or approximately 4 days in total. In the high latency (100 ms) networking setup **C**, it would take almost 17 days. We expect to drastically reduce this time in future work by adding record linkage blocking techniques to our procedure, which, for classical and Bloom filter-based record linkage, have already been implemented in recent versions of Mainzelliste. However, this setup would only need to run once initially, when two parties enter the secure record linkage system. Once the system is online and linked, securely linking a newly admitted patient to an existing database of size 10 000 would take 6.1 s online time for circuit variant GMW/A or 4 s in the pure GMW protocol, assuming network setting **A**. In network environment **C**, it would take 48 s for variant GMW/A. Also note the different scaling behaviors: due to the exhaustive pair comparisons, the computation- and communication complexity is O(M×N) during the *initial* linking phase, while during normal operation the complexity becomes O(N), i.e. linear in the size of the data source. This demonstrates the feasibility of our technique in a broad range of practical applications.

The optimal configuration of our system depends on the requirements of the scenario. For most environments, the optimization of the online times is sensible, as the setup phase can run between timing critical processes. For all cases other than having small databases and very high latencies, using variant GMW/A constitutes a sensible default. This allows non-technical personnel to deploy our system with a sensible configuration.

This work is easily generalizable to augmented patient data. If, for example, the IDAT fields used in this work were augmented by equality-check MDAT values, runtimes would not be impacted heavily. As displayed in Figure 4, simple equality comparisons are nearly negligible in comparison to fault-tolerant Bloom-Dice comparisons.

Our results are in alignment with Demmler et al.’s (2015a) observation that in most applications the utilization of mixed sMPC protocols is more efficient. The performance gains of the combined usage of GMW and Arithmetic Sharing outweigh the additional computations required for the conversions between the protocols.

The studied network environments reveal widely known bottlenecks of sMPC. Firstly, we can identify the network communication as the computation’s main impediment (cf. Fig. 3). By either throttling the network bandwidth or increasing the latency between both parties, runtimes significantly increase. A detailed analysis of the connection between the database sizes, network settings and circuit depths was given in Section 3.4. At least in Germany, this should not pose a strong impediment since research clinics are connected by the high-performance DFN network, which most closely resembles our best network environment **A**. We can also conclude that machines with more computational power would unfortunately not lead to significant improvements in runtime.

The legal question whether the transmitted data is ‘personal data’ is not answered yet in the European Union. Past decisions of the European Court of Justice and the German Federal Court of Justice lead to our understanding that record linkage without the patients consent might be legally permitted, as encrypted data is only personal data for parties having access to the encryption key as well as third parties having the legal right to demand disclosure of the key (Federal Court of Justice of Germany, 2017; The Court of Justice of the European Union, 2016). In the case that the encrypted data is seen as a pseudonym connected to additional information, the legal status is determined by the network (and availability) of the connected additional data. The referenced rulings have been made before the introduction of the European ‘General Data Protection Regulation (GDPR)’ (Eurpean Parliament and Council, 2016). We find it highly plausible that our record linkage solution indeed does not transmit personal data, but at the moment no legal verification of that claim is published.

## 5 Conclusion

In this work, we presented a novel method to perform privacy preserving record linkage with no information leakage, guaranteed by the utilization of provably secure multi-party computation. Most importantly, in the environment relevant for medical research in the foreseeable future (semi-honest setting and the absence of quantum computers), record linkage via MainSEL ensures that no record linkage party learns anything apart from the intended record linkage result—not even in an indirect (e.g. Bloom-filtered) form. Our implementation includes integration interfaces, optimizations and operation-ready deployment methods.

Due to carefully designed cryptographic protocols, as well as a novel high-level approach to generate optimized integer division circuits, our solution provides reasonable runtimes for linking mid-sized to large data sources as well as in an online mode for large and very large data sources. Albeit the promising results, this work opens up possibilities for further optimization and research in the following two categories: (i**)** the secure record linkage algorithms and (ii**)** the interfaces and application.

In practical applications, record linkage between more than two parties would be desirable but implies significant opportunities for research: probabilistic record linkage measures like ours are not transitive so in a multi-database setting, match conflicts may arise. In practice, such conflicts are usually resolved by accepting a non-direct-but-transitive match as a match, thereby interpreting such direct non-matches as false negatives. How to optimize network topologies to minimize linkage conflicts (e.g. with a star topology if feasible) is future research.

Note that we implemented a pair-wise record linkage algorithm using a secure *two*-party computation framework (ABY), so using MainSEL to fully link *k* databases of size *N*, each, requires the naive pairwise matching of $(k(k-1)N^{2})/2$ records. However, we’d like to stress that we built a drop-in replacement for the local record linkage that usually happens inside a single Mainzelliste instance in clear-text, so our solution can be readily deployed to the existing German medical research environment, where the Mainzelliste is in broad use, to enable novel research directions that would not be possible without a fully privacy-preserving linkage methodology. The usage of optimized algorithms for multi-database record linkage while using the *multi*-party successor to ABY will be explored in the future.

To further enhance the flexibility of the record linkage solution in this work, additional methods to include non-IDAT fields can be included. Those fields might lead to the need to include or develop different matching classifier. To reduce the computationally intensive areas of the procedure, it might be possible to include pruning and blocking methods. To be utilized, these must be provable secure and free of information leakage, which, in combination with sMPC protocols, presents an open research problem, as typical blocking techniques, such as *Locality Sensitive Hashing* based techniques, are shown to be incompatible to those strong privacy guarantees (He *et al.*, 2017). We plan to analyze the applicability of *Oblivious RAM*-based constructions in a potential blocking mechanism to avoid the degradation of our privacy guarantees.

Reliably deploying an application in the hospital IT environments is also a big challenge. The implementation of ICE techniques using STUN, TURN and other suitable methods for firewall and proxy traversal is the next step to be ready for hospital deployment. Our current implementation handles user authentication analogous to Mainzelliste. In the future, we want to provide OAuth2 and TLS client certificate authentication. Even though MainSEL is not designed as a secure record linkage software library, but as a generic interfaced standalone application, it could be adapted as a C++ Software Development Kit (SDK), or even software library, with moderate effort.

Independent of those areas of improvement, further regulatory and legal work is a necessary condition to allow practical usage of secure record linkage. With this work we hope to contribute to this process by providing practical benchmarks and technology details. In our opinion secure record linkage can contribute to a more privacy-preserving, better auditable and less bureaucratic digitized medicine.

We would like to stress, that even if secure record linkage is computationally intensive, many application scenarios become legally or intent-wise possible only through the privacy guarantees of our solution.

## Funding

This work was supported by the German Federal Ministry of Education and Research (BMBF) through the HiGHmed Consortium [01ZZ1802G]; and the German Research Foundation (DFG) through the MAGIC project [LA 3859/1-1] and the Research Training Group GRK 1651 (SOAMED).

*Conflict of Interest*: none declared.

## Supplementary Material

btaa764_Supplementary_Data
