# Supplementary material for: Mainzelliste SecureEpiLinker (MainSEL): privacy-preserving record linkage using secure multi-party computation
Source: Bioinformatics. 2020 Sep 1;38(6):1657–68. doi: 10.1093/bioinformatics/btaa764 (PMC8896632; doi:10.1093/bioinformatics/btaa764)
Supplement: btaa764_Supplementary_Data [file btaa764_supplementary_data.pdf]

Appendices

A Bloom Filters

A Bloom filter on a data set $x = \{x_j\}_{1 \le j \le m}$ is an encoding of x as a bit vector of fixed length l. All bits are initially set to 0. Now fix k independent hash functions $h_1, \ldots, h_k$. To store the data set x of size m into the Bloom filter, we apply all k hash functions to it and set the bits at the resulting positions $h_i(x_j) \mod l, 1 \le i \le k, 1 \le j \le m$. Denote the Bloom filter of a string x by $\mathrm{Bl}(x)$. Note that at most $k \cdot m$ bits are set this way and that it may well happen that a bit is set more than once, so this function is not injective. The collision probability is approximately (Mitzenmacher and Upfal, 2005)

$$\Pr(\mathrm{Bl}(x) = \mathrm{Bl}(y) \mid x \ne y) \approx \left(1 - e^{-km/l}\right)^k. \qquad (1)$$

The implementation of a Bloom filter using k independent hash functions can be improved drastically following the construction of Kirsch and Mitzenmacher (Kirsch and Mitzenmacher, 2006). Without an increase in the asymptotic false positive probability, k hash functions can be simulated using only two independent hash functions:

$$g_i(x) = h_1(x) + ih_2(x) \mod p; i = 0, \ldots, k-1, \qquad (2)$$

with p being the prime range of the hash functions. The Mainzelliste software uses $k = 15$ hash functions; using the optimization reduces the number of hashes calculated per bigram by a factor of 7.5.

q-grams. To get the Bloom filter of a string, it is first converted into such a data set x by splitting it into so called q-grams. The string is pre- and appended with a space and then split into sets of consecutive letters, ignoring case. The Mainzelliste software uses $q = 2$, in which case the string eva gets split into the 2-, or *bigrams*, {␣e, ev, va, a␣}. The hash functions are then applied to each q-gram.

B Record-Linkage Quality Analysis

B.1 Dataset Generation

For our quality analysis, we used synthetic datasets, generated with the Mockaroo synthetic data generation tool (https://www.mockaroo.com). As Mockaroo does not support all field types exactly as required, some post-processing of the raw datasets is required. For example the Mockaroo field type “Datetime” was split into the fields “day”, “month” and “year”. We generated a dataset with 50 000 records using the Mockaroo configuration in Table 1.

Table 1. The Mockaroo configuration used to create the raw dataset for the quality analysis of our implementations’ record linkage algorithm. The ZIP code range follows the German ZIP code scheme, with “01001” being the lowest issued code.

Field name	Type	Range
First Name	First Name (European)	
Surname	Last Name	
Birth name	Last Name	
Birth date	Datetime	01.01.1930 – 31.12.2019
ZIP code	Number	01001 – 99999
City	City	

As this configuration is not compatible with the state-of-the-art PPRL implementation of Lazrig *et al.* (2018) we generated a second dataset for the comparison, following their description. The Mockaroo parameters for this dataset are shown in Table 2. For this dataset 50 000 records were generated as well.

Table 2. The Mockaroo configuration used to create the raw dataset for the quality comparison between our implementation’s record linkage algorithm and that of Lazrig et al. (2018). The configuration follows Lazrig et al.’s dataset structure.

Field name	Type	Range
First Name	First Name (European)	
Surname	Last Name	
Birth date	Datetime	01.01.1930 – 31.12.2019
SSN	SSN	

Data Selection and Perturbation. The generated raw datasets need to be further processed to prepare them for the record linkage quality analysis. First, N records are randomly sampled, where N denotes the overall number of required unique records. Then, 60 % of “birth name” fields are removed, as this field is often empty in realistic datasets. This step is omitted in the comparison with Lazrig *et al.*, as they do not use a “birth name” field and additionally cannot handle empty fields. The resulting set is then split into two datasets with respective sizes N_1 and N_2. The second dataset is constructed so that it has an adjustable overlap with dataset 1 and its records are randomly shuffled.

Every field in dataset 2 is stochastically permuted. With equal probability the following permutations are applied:

1. the deletion of a random symbol in the field,
2. the exchange of two random symbols in the field,
3. the field is set to empty.

After this field-wise perturbation, the fields in a record are shuffled with the same perturbation probability. Shuffling is only applied to compatible fields, e.g., first name and surname, or birth day and birth month. Shuffling between, e.g., the city name and the birth year is not permitted, as the fields contain different data types. The modifications are not singular, but each field and record can have multiple perturbations. Finally, the dates are sanitized, such that only valid day/month combinations are generated. The probability of the individual modifications is calculated such that an adjustable overall field perturbation probability is achieved. This is done to be comparable to Lazrig *et al.*’s analysis method. Again, the emptying of a field is omitted in the comparison with their work.

B.2 Quality of Record Linkage

The two datasets used for the evaluation of our EpiLink implementation are generated following the procedure described in Sec. B.1. Both datasets contain 10 000 records with an overlap of 60 %. The probability of an empty “birth name”-field is set to 60 %. The probability of each individual perturbation is set to 2.6 % and 5.4 %, as those values correspond to a field perturbation probability of respectively 10 % and 20 %. The configuration of the EpiLink algorithm is stated in Table 5 in Section D. All benchmarks were performed with a bit precision of $L = 32$ bit. The results are shown in Table 3, where “TP”, “FP” and “FN” denote “True Positives”, “False Positives” and “False Negatives”, respectively. “Recall” is the True Positive Rate $R = \frac{TP}{TP+FN}$ and “Precision” is $P = \frac{TP}{TP+FP}$. The “F_1-Score” and “Matthews Correlation Coefficient” (MCC) are combined evaluation

metrics for binary classification defined as follows:

$$F_1 = \frac{2TP}{2TP+FP+FN}$$
$$MCC = \frac{TP \times TN - FP \times FN}{\sqrt{(TP+FP)(TP+FN)(TN+FP)(TN+FN)}}$$

Table 3. Record linkage results using our EpiLink implementation. The error rate is per field. Each dataset has 10 000 records with 60 % overlap between sets. “TP”, “FP” and “FN” denote “True Positives”, “False Positives” and “False Negatives”, respectively. The “F_1-Score” and “Matthews Correlation Coefficient” (MCC) are combined evaluation metrics for binary classification.

Error Rate	TP	FP	FN	Recall	Precision	F_1	MCC
0.1	5967	0	33	0.994	1.0	0.997	0.993
0.2	5820	4	179	0.970	0.982	0.984	0.963

To compare the record linkage quality of our solution to the state-of-the-art solution, we implemented the algorithm of Lazrig *et al.* (2018). We followed the description in their publication by generating Bloom filters over the following combinations:

1. First name + Last name + Date of Birth,
2. Date of Birth + SSN,
3. Last name + SSN,
4. Three letters first name + Three letters last name + Soundex first name+ Soundex last name + Date of birth + SSN.

Furthermore, we used the same parameters as published by Lazrig *et al.* for the evaluation of their algorithm, that is, Bloom filters of length 1000 bit, 30 hash-functions and a random salt. However, we did not implement differentially private blocking and their Bloom filter partitioning scheme, which would result in a lower accuracy but better runtime performance. For the evaluation of our EpiLink implementation, we used the parameters from Table 5 for every applicable field. For field “SSN” we chose a frequency of 10×10^{-9} (assuming uniformly i.i.d. digits in the SSN) and an error rate of 0.088—the same as for the first name. All Bloom filters are 500 bit long, using 15 hash-functions.

The dataset for this evaluation has fewer valid perturbations, as empty fields are not allowed in Lazrig *et al.*’s work, so the individual modification probability is adjusted for the chosen field perturbation probability. As both algorithms perform very well, it was necessary to choose a very high field perturbation probability to properly evaluate the quality differences. Consequently, we chose a field perturbation probability of 40 %. The results for the linkage of two datasets with each 10 000 entries and 60 % overlap are shown in Table 4.

Table 4. Comparison of record linkage quality between the state-of-the-art algorithm of Lazrig et al. and the EpiLink implementation in this work. The error rate is per field is set to 40 %. Each dataset has 10 000 records with 60 % overlap between sets. “TP”, “FP” and “FN” denote “True Positives”, “False Positives” and “False Negatives” respectively. The “F_1-Score” and “Matthews Correlation Coefficient” (MCC) are combined evaluation metrics for binary classification.

	TP	FP	FN	Recall	Precision	F_1	MCC
Lazrig *et al.*	5917	2	63	0.989	0.999	0.993	0.986
This work	5970	1	31	0.995	0.999	0.997	0.993

C Secure Two-Party Computation

C.1 Generic sMPC Protocols and Secret Sharing

The crucial observation of early sMPC works (Yao, 1986; Goldreich *et al.*, 1987) is that *any* efficiently computable functionality f can be implemented as a secure sMPC protocol. However, there are many ways in which this can be achieved. Here, we describe the three approaches implemented by the ABY framework.

Yao’s Garbled Circuit. This method, introduced by Yao (Yao, 1986), assigns two roles to the parties performing the sMPC: A *Garbler* and an *Evaluator*. Given a Boolean circuit encoding the desired functionality, consisting of only AND and XOR gates, the Garbler encrypts it in a gate-by-gate fashion, and sends this garbled version to the Evaluator, along with their garbled inputs. The parties then perform an Oblivious Transfer (cf. Section C.2) which allows the Evaluator to receive their own garbled inputs and evaluate the circuit. This protocol requires a low constant number of communication rounds. For a full description and a security proof, see (Lindell and Pinkas, 2009).

GMW. Named after its inventors Goldreich, Micali and Widgerson (Goldreich *et al.*, 1987), this protocol also represents the functionality as a Boolean circuit. Intermediate values are represented as *XOR shares*, i.e., for each bit b in the shared value, each party $i \in \{0, 1\}$ holds a uniformly random bit b_i, such that $b = b_0 \oplus b_1$. This is analogous to b_1 being a *One Time Pad* for bit b_0. Now, the parties evaluate the circuit in parallel gate-by-gate: XOR gates are evaluated locally by simply XORing the corresponding shares, and AND gates are computed in an interactive protocol involving Oblivious Transfer (see Section C.2). Because of this interactivity, the required number of communication rounds is equal to the depth of the circuit.

Arithmetic Sharing. Here, the computation is represented using an *arithmetic circuit* that consists of multiplication and addition gates. Similarly to GMW, intermediate values are stored as random shares that add up to the actual value. Additions can then be performed locally, while multiplications are done using a dedicated sub-protocol such as Gilboa multiplication (Gilboa, 1999). As for GMW, the required number of communication rounds is equal to the depth of the circuit.

Apart from the three sMPC protocols above, ABY also implements methods for converting intermediate values between them.

C.2 Oblivious Transfer

All three protocols described in the previous section rely on a cryptographic primitive called Oblivious Transfer (OT). Its mechanism was introduced by Wiesner (Wiesner, 1983) under the name “Conjugate Coding” and popularized by Rabin (Rabin, 2005). The idea is that one party, the *sender*, prepares two messages m_0, m_1 and the second party, the *receiver*, chooses which message to receive with a choice bit c, without the sender learning which message was chosen and without the receiver learning anything about the other message.

Recent improvements made OT suitable for practical sMPC (Asharov *et al.*, 2013). OT Extensions (Ishai *et al.*, 2003; Asharov *et al.*, 2017) for example use a small number of (rather expensive) “base” OTs to cheaply generate a large number of following OTs, thus reducing the required number of computationally expensive cryptographic operations drastically.

C.3 ABY Security Assumptions and Guarantees

Independent of the circuit design, each ABY sMPC starts with the generation of base-OTs in the setup phase. ABY implements a protocol from Naor *et al.* (2001), whose security is guaranteed under the Computational Diffie-Hellman (CDH) hardness assumption. CDH is strongly related to the discrete logarithm problem and would be broken by Shor’s algorithm

on quantum computers. The protocol also relies on the random oracle assumption, for which ABY uses the SHA256 hash function for our selected security parameters. After computation of the base-OTs, the actual OTs are calculated by OT Extensions using fixed-key AES as a random permutation source. This is still assumed to be secure in our semi-honest setting, but has recently been shown to be theoretically insecure in the malicious setting (Guo *et al.*, 2019). When using Yao's Garbled Circuit, the same security assumption on a random permutation source applies. ABY guarantees information-theoretical security for XOR and arithmetic sharing of inputs.

D Field Configuration

Table 5. The default EpiLink field configuration of the DKFZ Mainzelliste, which was also used in the reported benchmarks. The Comparison is either "Equality" (Eq.) or "Bloom-Dice" (B.D.). The weight w is calculated as $w = \log\left({}^{(1-e)}/_{f}\right)$.

Field name	Type	Comparison	Frequency f	Error Rate e	Weight w	Bitlength
First Name	String	B.D.	0.000 235	0.01	12.04	500
Surname	String	B.D.	0.000 027 1	0.008	15.16	500
Birth name	String	B.D.	0.000 027 1	0.008	15.16	500
Day of birth	Integer	Eq.	0.0333	0.005	4.90	5
Month of birth	Integer	Eq.	0.0833	0.002	3.58	4
Year of Birth	Integer	Eq.	0.0286	0.004	5.12	11
ZIP code	String	Eq.	0.01	0.04	6.58	40
City	String	B.D.	0.01	0.04	6.58	500

E Benchmark Tables

Table 6. Comparison of the setup and online runtimes for the three networking configurations from Figure **??**, for varying database sizes for the four circuit protocol variants GMW, GMW/A, Yao and Yao/A.

Database	Comm. [MiB]			Setup Phase [s]			Online Phase [s]		
Size	#Rounds	Setup	Online	A	B	C	A	B	C
GMW circuit variant									
1	370	2.7	0	0.047	0.1	0.85	0.11	0.17	19
10	530	25.2	0.4	0.19	0.42	1.5	0.15	0.24	27
25	570	62.5	1	0.35	1.9	2.1	0.17	0.24	29
100	650	248.8	3.9	1.2	10	5.5	0.23	0.37	33
250	690	621.2	9.8	3	27	12	0.32	0.48	36
1,000	770	2,483.4	39.3	12	110	44	0.63	1.7	40
2,500	850	6,207.9	98.3	29	270	110	1.3	4.2	45
10,000	930	24,830.6	393	120	1,100	450	3.9	17	53
25,000	970	62,076.2	982.5	300	2,700	1,100	8.8	44	66
GMW circuit variant with arithmetic conversions									
1	266	0.6	0.1	0.018	0.036	0.72	0.052	0.054	13
10	330	5.5	0.7	0.097	0.15	1.4	0.072	0.072	16
25	346	13.5	1.7	0.18	0.29	1.6	0.093	0.094	17
100	378	53.7	6.7	0.43	1.7	2.5	0.17	0.17	18
250	394	133.9	16.8	0.87	5.3	4	0.29	0.3	19
1,000	426	555.2	47.1	3	23	11	0.77	0.87	22
2,500	458	1,394.1	119.5	7.3	60	25	1.6	1.9	27
10,000	490	5,577.4	459.4	28	240	96	6.1	8.2	48
25,000	506	13,917.9	1,150.3	69	610	240	15	23	88
Yao circuit variant									
1	5	0	2.4	0.055	0.09	0.15	0.065	0.092	0.85
10	5	20.3	3.8	0.26	0.3	0.67	0.2	0.34	1.8
25	5	52.8	7.6	0.61	0.61	1.1	0.38	0.7	2.8
100	5	227.3	42.3	2.1	2.2	2.5	1.2	2.5	8.7
250	5	576.8	119.2	4.9	4.6	5.2	2.9	6.4	20
1,000	5	1,905.1	558.8	17	19	19	14	25	76
2,500	5	4,762.1	1,430.8	43	52	44	35	64	190
10,000	5	19,538.8	5,729.7	170	200	170	140	280	750
Yao circuit variant with arithmetic conversions									
1	40	0.1	0.6	0.017	0.033	0.4	0.022	0.026	1.7
10	76	1.2	5.8	0.084	0.1	0.94	0.099	0.11	3.1
25	85	11.1	6.4	0.18	0.21	1.2	0.16	0.2	3.5
100	103	52.3	17.7	0.63	0.7	2	0.42	0.59	5.4
250	112	139	40.2	1.3	1.9	3	0.93	1.3	8.6
1,000	130	554.4	231.1	4.6	10	7	3.8	5.9	24
2,500	148	1,412.2	546.2	12	27	16	9	16	54
10,000	166	4,860.8	2,285.6	42	110	60	39	67	200

References

Asharov, G., Lindell, Y., Schneider, T., and Zohner, M. (2013). More efficient oblivious transfer and extensions for faster secure computation. In *Proceedings of the 2013 ACM SIGSAC Conference on Computer & Communications Security - CCS '13*, pages 535–548, Berlin, Germany. ACM Press.

Asharov, G., Lindell, Y., Schneider, T., and Zohner, M. (2017). More Efficient Oblivious Transfer Extensions. *Journal of Cryptology*, **30**(3), 805–858.

Gilboa, N. (1999). Two Party RSA Key Generation. In *CRYPTO*, volume 1666 of *Lecture Notes in Computer Science*, pages 116–129. Springer.

Goldreich, O., Micali, S., and Wigderson, A. (1987). How to Play any Mental Game or A Completeness Theorem for Protocols with Honest Majority. In *STOC*, pages 218–229. ACM.

Guo, C., Katz, J., Wang, X., and Yu, Y. (2019). Efficient and Secure Multiparty Computation from Fixed-Key Block Ciphers. Technical Report 074, Cryptology ePrint Archive.

Ishai, Y., Kilian, J., Nissim, K., and Petrank, E. (2003). Extending Oblivious Transfers Efficiently. In *CRYPTO*, volume 2729 of *Lecture Notes in Computer Science*, pages 145–161. Springer.

Kirsch, A. and Mitzenmacher, M. (2006). Less Hashing, Same Performance: Building a Better Bloom Filter. In Y. Azar and T. Erlebach, editors, *Algorithms – ESA 2006*, Lecture Notes in Computer Science, pages 456–467. Springer Berlin Heidelberg.

Lazrig, I., Ong, T. C., Ray, I., Ray, I., Jiang, X., and Vaidya, J. (2018). Privacy Preserving Probabilistic Record Linkage Without Trusted Third Party. In *2018 16th Annual Conference on Privacy, Security and Trust (PST)*, pages 1–10.

Lindell, Y. and Pinkas, B. (2009). A proof of security of yao's protocol for two-party computation. *J. Cryptology*, **22**(2), 161–188.

Mitzenmacher, M. and Upfal, E. (2005). *Probability and computing: Randomized algorithms and probabilistic analysis*. Cambridge university press.

Naor, M., Pinkas, B., and Pinkas, B. (2001). Efficient Oblivious Transfer Protocols. In *Proceedings of the Twelfth Annual ACM-SIAM Symposium on Discrete Algorithms*, SODA '01, pages 448–457, Philadelphia, PA, USA. Society for Industrial and Applied Mathematics.

Rabin, M. O. (2005). How to Exchange Secrets with Oblivious Transfer. *IACR Cryptology ePrint Archive*, **2005**, 187.

Wiesner, S. (1983). Conjugate coding. *ACM SIGACT News*, **15**(1), 78–88.

Yao, A. C.-C. (1986). How to Generate and Exchange Secrets (Extended Abstract). In *FOCS*, pages 162–167. IEEE Computer Society.
